# 71 Enhancing physical activity through service design

**DOI:** 10.1093/eurpub/ckae114.133

**Published:** 2024-09-26

**Authors:** Sanna Kangas, Liisa Kiviluoto-Heinonen

**Affiliations:** LAB University Of Applied Sciences, Lahti, Finland; Haaga-Helia University Of Applied Sciences, Heinola, Finland

## Abstract

**Purpose:**

The purpose of LIPA (Users as the developers of sport services) project (implementation in Päijät-Häme region, Finland) was to help companies to develop sport services that meet the customer’s needs, which helps to reach new customer groups. Finding out customers’ needs also helps the companies to find customers that they haven’t reached yet, and this way enhance leisure time physical activity of new target groups.

**Project description:**

LIPA project produced new information, understanding and know-how about the user-oriented development of sports services for companies in the sports and well-being sector. In the project, together with 10 sports companies, existing or completely new services were developed using service design.

For the development of the services, customer understanding was collected from existing and potential customers. Based on customer understanding, customer personas were crystallized, through which new or improved services could be conceptualized. In addition to experts and entrepreneurs, customers or a possible future user group also participated in the ideation and piloting. Each development process was evaluated from the perspective of the project team and the entrepreneur utilizing the RE-AIM framework.

Through the service design processes, new and improved services were created, which are aimed at new users and customer groups. As a result, the described model service design process is also created - a model for user-oriented development of sports services and the user persona of sports services in the private sector. The resulting sports service design process model will be disseminated to be used by the sports industry in cooperation with the higher education network and entrepreneurs.

**Conclusions:**

With the means of service design, it is possible to identify the needs of target groups that were previously perceived as challenging, and it is also possible to take these needs into account when communicating and marketing services. Service design also enables the development of services in a customer-oriented manner, and newly developed or redesigned services meet the needs of users better. This could be a possibility to increase leisure time activity even for those who are less physically active.

